# Hyperglycaemia-induced molecular reprogramming of proximal tubular epithelial cells and its contribution to diabetic kidney disease progression—a narrative review

**DOI:** 10.3389/fcell.2026.1913927

**Published:** 2026-09-10

**Authors:** Terry Gbaa

**Affiliations:** 1 Margaret Lawrence University College of Medical Sciences, Abuja, Nigeria; 2 JB Consulting MDP Limited, Berkshire, United Kingdom

**Keywords:** biomarkers, diabetic kidney disease (DKD), hyperglycaemia, molecular reprogramming, proximal tubular epithelial cells (PTECs), renal tubulopathy, therapeutic targets

## Abstract

Diabetic kidney disease (DKD) is the primary contributor to the development of chronic kidney disease and end-stage kidney failure globally. A hyperglycaemic microenvironment is a critical contributor of proximal tubular epithelial cell injury to disease initiation and progression. Hyperglycaemia induces profound molecular, metabolic, and structural alterations in renal tubular epithelial cells, promoting inflammation, fibrosis, and functional decline. Chronic hyperglycaemia activates multiple pathogenic pathways, including the polyol, hexosamine, protein kinase C (PKC), and advanced glycation end-product pathways, resulting in excessive reactive oxygen species generation, mitochondrial dysfunction, inflammation, and extracellular matrix accumulation. Hyperglycaemia also alters the expression of key genes, including *biglycan*, advanced glycation end products receptor, and BCL2 interacting protein 3, contributing to cellular dysfunction and disease progression. Emerging evidence supports the role of extracellular vesicles, exosomal RNAs, and mitochondrial nucleic acids in mediating tubular injury. Advanced diagnostic platforms, including urinary biomarker panels, proteomics, single-cell RNA sequencing, spatial transcriptomics, and multi-omics approaches, offer improved sensitivity for early detection and mechanistic characterisation of DKD. A hyperglycaemic microenvironment could damage the proximal tubular epithelial cells via mechanisms such as dedifferentiation, metabolic reprogramming, cell death and senescence and other pathways (ENPP1, HIF stabilisation and epigenetic changes). Therefore, these mechanisms are the targets of emerging and novel therapeutic strategies which include HIF-Prolyl Hydroxylase Inhibition, Targeting Maladaptive SOX9^+^ Cells, Metabolic Targets, Senescence and Senolytics/Senomorphics.

## Graphical Abstract

**Figure:**
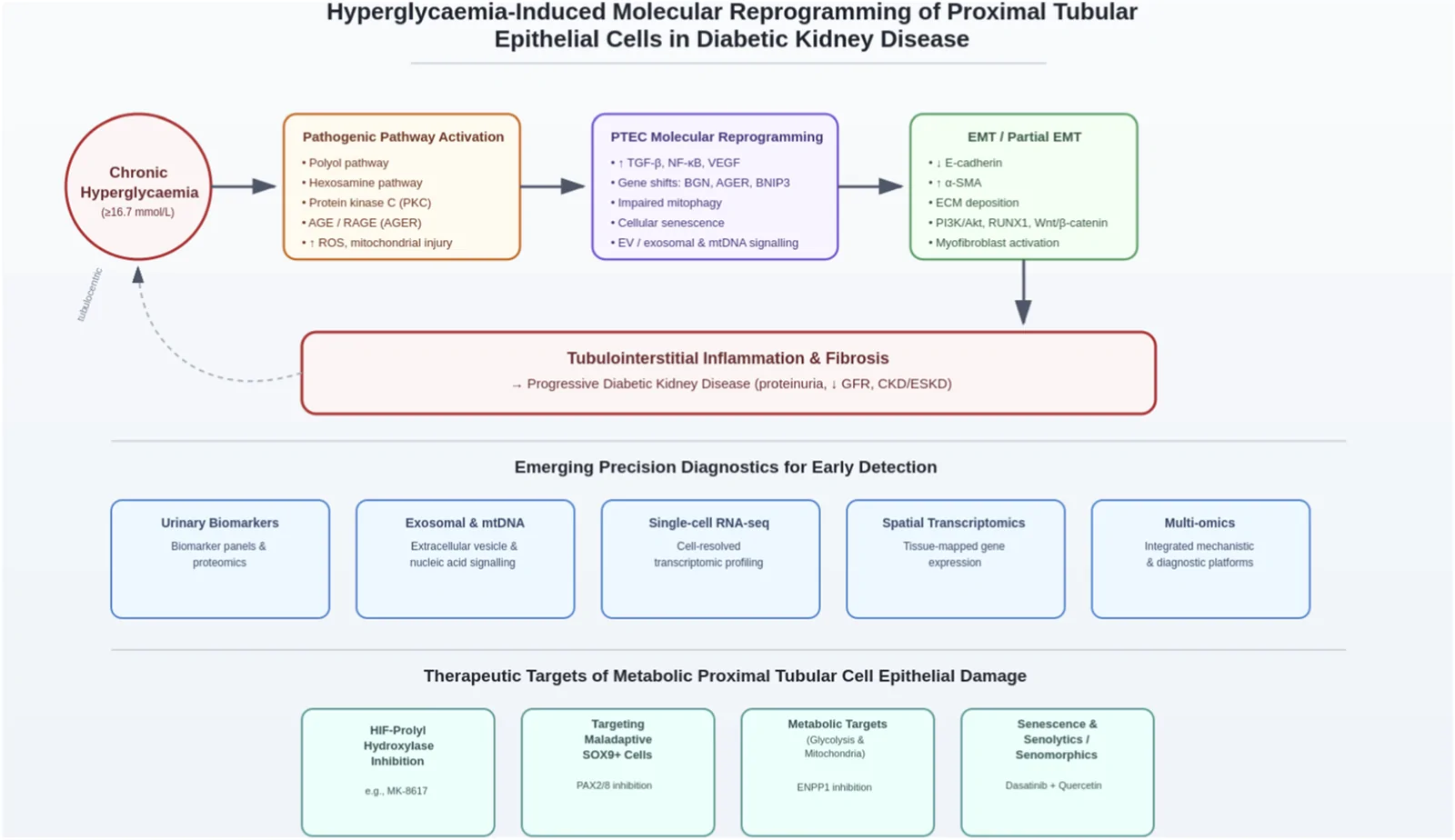

## Introduction and background

Hyperglycaemia (high blood glucose levels) has been associated with adverse effects on renal tubular cells and has been proposed to cause damage to the tubular cell linings in the kidneys, resulting in functional impairments and disruptions (Wang et al., 2023). This contributes to the loss of absorptive capacity, alterations in urinary protein handling, or changes in biomarker profiles. There is currently a shift away from the traditional glomerular-centric view of diabetic kidney disease (DKD) toward a more “tubulocentric” understanding of renal dysfunction.

The elevation of glucose concentrations directly correlates with an amplified build-up of reactive oxygen species within tubular epithelial cells; as a result, this increases the cascade of inflammatory damage (Wang et al., 2020). Hyperglycaemic conditions enhance reactive oxygen species (ROS) production, cellular senescence pathways, and induction of epithelial–mesenchymal transition, in which cells acquire mesenchymal characteristics and promote extracellular matrix deposition (Wang et al., 2023; Xu et al., 2024). The renal extracellular matrix (ECM) is a dynamic structure that can undergo remodelling, especially during fibrosis, and these ECM proteins exhibit cross-linking, making them more stable and less degradable (Vallon and Komers, 2011).

Chronic hyperglycaemic conditions lead to the activation of various pathways, including the polyol and hexosamine pathways, and the accumulation of advanced glycation end-products. These pathways initiate a series of events involving different second messengers, such as transforming growth factor β-1 (TGF-β1), nuclear factor-κB, and vascular endothelial growth factor, which regulate critical cellular processes and contribute to the development and progression of DKD (Tao et al., 2022).

Hyperglycaemia is associated with increased expression of IL-1α, fibronectin, α-SMA, and other ECM proteins and profibrotic cytokines (Salti et al., 2020). High glucose levels increase the mRNA expression of BCL2 interacting protein 3 (BNIP3) in renal tubular epithelial cells (Huang et al., 2016). Understanding the behavioural patterns and molecular profiles of renal tubular cells exposed to increased glucose concentration would offer more insight into tubulointerstitial fibrosis and the development and progression of DKD. Recent diagnostic advances utilise a combination of analytical tools, including urine biomarker panels, exosomal RNAs, proteomics, and spatial transcriptomic maps, to improve sensitivity and mechanistic specificity (Lake et al., 2023; Zhang and Parikh, 2019).

This narrative review aims to expand current knowledge on the effects of hyperglycaemia on the molecular reprogramming of proximal tubular epithelial cell function, with particular emphasis on the molecular and cellular mechanisms underlying renal tubular injury. Additionally, this review also looks at the translational and transcriptomic advances in the diagnosis of renal tubular epithelial cell dysfunction associated with hyperglycaemia. By synthesising recent evidence on pathways involved in oxidative stress, mitochondrial dysfunction, inflammation, autophagy, and cellular senescence, this review seeks to provide clinicians and researchers with a comprehensive understanding of the effect of hyperglycaemia on renal tubular epithelial cells and the progression of DKD. Such insights may facilitate the development of more effective strategies for early diagnosis, risk stratification, and biomarker discovery.

## Methodology

A comprehensive literature search was conducted in PubMed, Scopus, Embase, and Web of Science from database inception to March 2025. Search terms included combinations of “diabetic kidney disease”, “renal tubular injury”, “glomerular filtration barrier”, “mitophagy”, “autophagy”, “fibrosis”, “epithelial-mesenchymal transition”, “oxidative stress”, “biomarkers”, “single-cell RNA sequencing”, “spatial transcriptomics”, and “kidney regeneration”. Boolean operators (AND/OR) were applied to optimise retrieval. Additional studies were identified through manual screening of reference lists of eligible articles and International Diabetes Federation publications. Duplicate records were removed before title and abstract screening.

**FIGURE 1:**
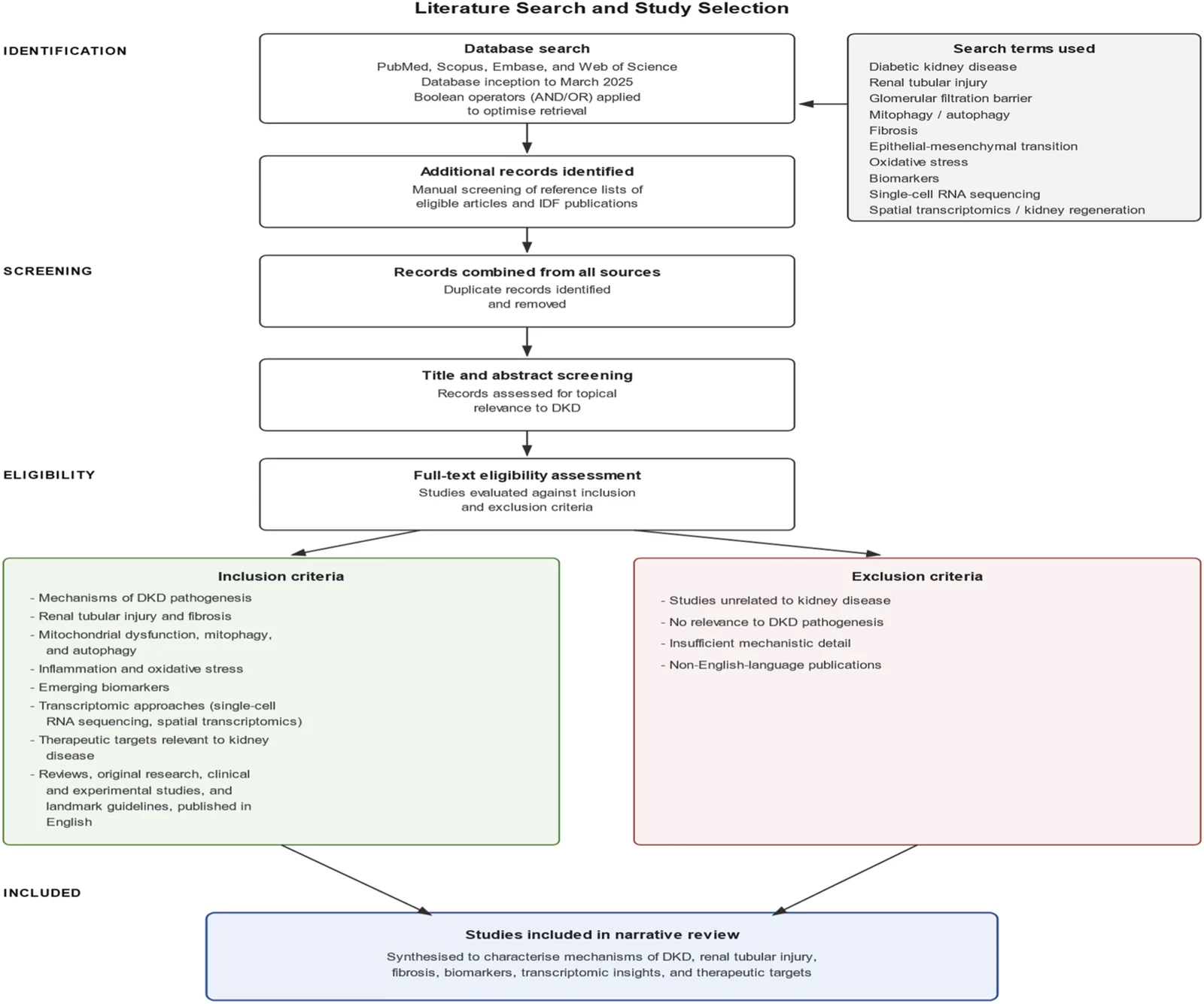
Flowchart for literature search and study selection. This is a comprehensive literature search and study selection for identification, screening and eligibility included in this narrative review.

Studies were included if they investigated mechanisms of DKD, renal tubular injury, fibrosis, mitochondrial dysfunction, inflammation, emerging biomarkers, transcriptomic approaches, or therapeutic targets relevant to kidney disease. Reviews, original research articles, clinical studies, experimental investigations, and landmark guideline documents published in English were considered. Studies unrelated to kidney disease, lacking relevance to DKD pathogenesis, or containing insufficient mechanistic information were excluded. The flowchart was adapted to suit a narrative review, and the literature search and the inclusion and exclusion criteria were included for transparency (Figure 1).

## Hyperglycemia and renal cell signalling: polyol, hexosamine, PKC, and age signalling in proximal tubular epithelial cells

Hyperglycaemia (high blood glucose levels) is the hallmark of diabetes mellitus and has been associated with adverse effects on proximal tubular epithelial cells. It has been proposed that a glucose concentration of 16.7 mmol/L causes damage to the tubular cell linings in the kidneys, resulting in functional impairments and disruptions (Wang et al., 2020). These effects result in persistent albumin loss (>300 mg/24 h) or a urine albumin-creatinine ratio exceeding 300 mg/g in at least two of three consecutive samples (Zhu et al., 2020; Wolf et al., 1993). Furthermore, the elevation of glucose concentrations directly correlates with an amplified build-up of reactive oxygen species within tubular epithelial cells (Wang et al., 2020); as a result, the cascade of inflammatory damage is increased. Another significant mechanism contributing to proximal tubular epithelial cell (PTEC) damage is the inhibition of mitophagy during acute hyperglycaemia (Wang et al., 2020). Mitophagy is the process of removing damaged mitochondria through lysosomal degradation (Bülow and Boor, 2019). This is the foundation of hyperglycaemic effects on renal cell signalling, which causes a reprogramming of tubular epithelial cells, especially the proximal cells which are prone to damage by the effect of glucotoxicity.

Several factors have been identified as influencers of proximal tubular cell proliferation. For instance, fatty acids can induce excessive proliferation, potentially resulting in the senescence of proximal tubular cells (Lin et al., 2016). This concept could be attributed to the lipotoxicity mechanism of diabetes (Brings et al., 2015). Additionally, inhibitors of the renin-angiotensin system have demonstrated the ability to reduce ECM accumulation, which indicates that locally produced angiotensin II by proximal tubular cells promotes ECM accumulation (Gronda et al., 2020). The renal ECM is a dynamic structure that can undergo remodelling, especially during fibrosis (Gerich, 2009). It consists of various proteins, including glycoproteins and proteoglycans, as well as structural markers that change expression during fibrosis. These ECM proteins undergo cross-linking during fibrosis, making them more stable and less degradable (Pasupuleti et al., 2020). This is the basis of scaffolding for advanced glycation end-products in DKD (Darwish et al., 2020).

PTECs play a crucial role in regulating glucose metabolism by contributing to glucose production through gluconeogenesis (Bayram and Kızıltan, 2024). They utilise approximately 10% of the body’s total glucose (Naaman and Bakris, 2023). However, when the standard mechanisms maintaining glucose balance are disrupted, elevated glucotoxicity increases oxidative stress in tubular cells (Dou and Jourde-Chiche, 2019). This oxidative stress results from activating various pathways, including the polyol and hexosamine pathways, and the accumulation of advanced glycation end products (Vallon and Komers, 2011).

These pathways initiate a series of events involving different second messengers, including PKC, MAP kinase, and nuclear transcription factors like TGF-β1, nuclear factor-κB (NF-κB), and vascular endothelial growth factor (VEGF) (Vallon and Komers, 2011). Activating these second messengers orchestrates a complex network of intracellular events, leading to the modulation of cellular functions and the regulation of critical biological responses. For example, these cascades are crucial in transmitting signals within the cell, regulating critical cellular processes, and influencing the expression of genes such as advanced glycation end products receptor (AGER) and biglycan (BGN) in proximal tubular cells. This contributes to the progression of tubular dysfunction. Hyperglycaemia triggers an upregulation of nuclear transcription, releasing inflammatory cytokines and leading to an inflammatory milieu (Chung et al., 2015). Understanding the complexities and drivers of proximal tubular cell growth and damage can enhance our knowledge of diabetes and DKD. As a result, this may facilitate advancements in current treatment techniques for acute and chronic renal illnesses.

## Transcriptional and phenotypic reprogramming of PTECs

We have established that the hyperglycaemia and renal cell signalling show that polyol/hexosamine pathway activation alters PTEC metabolism. We examined how this metabolic stress is transduced into the transcriptional reprogramming that defines the EMT/pEMT phenotype (epithelial–mesenchymal transition) (EMT) is a process involved in the pathogenesis of renal fibrosis. This common mechanism leads to end-stage renal failure (Lovisa et al., 2015). EMT involves transforming renal tubular epithelial cells into mesenchymal-like cells, acquiring fibroblast characteristics, and promoting ECM deposition (Hills et al., 2012).

EMT is characterised by the loss of epithelial markers, such as E-cadherin, and the acquisition of mesenchymal markers, such as α-smooth muscle actin (α-SMA) (Hills et al., 2012; Shen et al., 2022). Several signalling pathways have been implicated in the induction of EMT and renal fibrosis, such as Phosphoinositide 3-kinase and protein kinase B signalling pathway (PI3K/Akt) signalling. The TGF-β pathway is a crucial regulator of EMT and is involved in activating various downstream signalling cascades, including the PI3K/Akt/Mammalian target of rapamycin (mTOR) pathway (Lovisa et al., 2015; Hsieh et al., 2014).

Runt-related transcription factor 1 (RUNX1) has been identified as a TGF-β-induced EMT and renal fibrosis promoter through the PI3K subunit p110δ, which mediates Akt activation (Lovisa et al., 2015). The Wingless and Int-1/beta-catenin signalling pathway (Wnt/β-catenin) pathway has also been implicated in renal fibrosis induced by chronic exposure to the herbicide atrazine (Shen et al., 2022).

Emerging research has provided insight into partial epithelial–mesenchymal transition (pEMT), which challenges the traditional view of complete EMT in renal fibrogenesis. ‘pEMT’ refers to renal tubular epithelial cells acquiring mesenchymal characteristics without detachment from the basement membrane (Hills et al., 2012; Nastase et al., 2014). This concept suggests that renal tubular epithelial cells can contribute to renal fibrogenesis by acquiring mesenchymal characteristics and producing profibrotic factors and cytokines while remaining attached to the renal basement membrane (Hills et al., 2012). The cellular response of epithelial cells to injury and the presence of transforming growth factor-beta 1 (TGF-β1) can result in cell arrest, impaired repair, and compromised regeneration. These effects are associated with the activation of myofibroblasts through the release of cytokines by partial epithelial-to-mesenchymal transition (EMT) cells attached to the basement membrane.

A study by Yamagishi et al. (2015) demonstrated that hyperglycaemia increased intracellular reactive oxygen species (ROS) levels and induced EMT through TGF-β1 in HK-2 cells. Another study revealed that hyperglycaemic conditions in renal tubular epithelial cells enhanced ROS production, resulting in DNA damage and activation of cellular senescence pathways (Forbes et al., 2008).

The kidney exhibits diverse targets within its extracellular matrix, which could be upregulated or downregulated. One such target is biglycan (BGN), a small leucine-rich repeat proteoglycan (SLRP) found in various organs. The *BGN* gene encodes this protein; BGN is primarily expressed in the tubulointerstitium of the renal cortex as well as in other kidney components such as peritubular mesenchymal cells and glomerular epithelial cells (Yamazaki et al., 2021).

**FIGURE 2:**
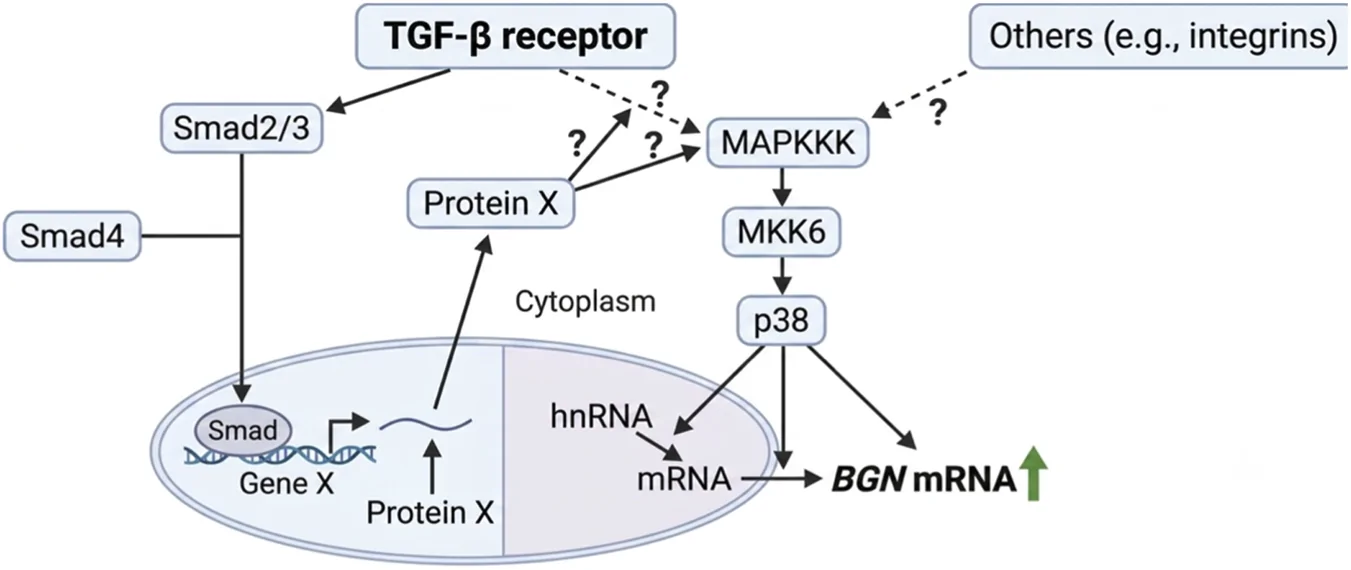
The role and mechanism of TGF-β and the upregulation of BGN mRNA (Ungefroren et al., 2003). TGF-β engages with Smad, inducing a direct stimulation of MAPKKK via protein X, subsequently initiating the transformation of hnRNA into mRNA through P38 enzymatic activity (hnRNA-heterogeneous nuclear *ribonucleic acid; MKK6: Mitogen-activated protein kinase 6*, *Mitogen-activated protein kinase kinase)*.

BGN mRNA is detectable in tubulointerstitial peritubular fibroblasts, distal tubules, collecting ducts, endothelial cells, smooth muscle cells, and blood vessel adventitia (Yamazaki et al., 2021). Expression of BGN can be linked to kidney diseases. Notably, it has been observed that TGF-β stimulates the expression of BGN mRNA. Increased circulation of biglycan is noticeable in several pro-inflammatory renal diseases (Cheng et al., 2015) (Figure 2).

Another target is AGER found in the kidneys. This receptor is also expressed on cell surfaces and interacts with various ligands, including advanced glycation end products (AGEs), proteins from the S100/calgranulin family, and high-mobility group box 1 (HMGB1). In DKD, the *AGER* gene shows increased expression. Elevated levels of these ligands are typical in individuals with diabetes and contribute to the inflammatory and oxidative stress reactions associated with DKD (Hu et al., 2024).

Activation of RAGE initiates pathways that stimulate inflammation, triggering the release of cytokines and chemokines. Chronic inflammation significantly contributes to the deterioration of renal cells and the progression of DKD. RAGE activation also induces oxidative stress, leading to the production of reactive oxygen species (ROS). Elevation of ROS levels is associated with detrimental effects on renal cells, encompassing cellular damage, inflammation, and fibrosis (Yao et al., 2018).

The expression of *AGER* is instrumental in the accumulation of extracellular matrix proteins within the renal system, ultimately leading to fibrosis. This process further impairs renal function and can lead to the emergence of conditions like glomerulosclerosis and tubulointerstitial fibrosis (Yao et al., 2018).

Hyperglycaemia is associated with upregulating the expression of ECM proteins in tubular cells. Studies have reported the upregulation of HMGB1 expression in hyperglycaemic conditions in renal tubular cells (Lv et al., 2017; Gu et al., 2020). HMGB1 activates Toll-like receptors (TLRs), leading to NF-κB translocation and the subsequent production of proinflammatory cytokines (Slezak et al., 2024; Li et al., 2023). TGF is also implicated in the disruption and disorganisation of tubular and mesangial cells (Ståhl et al., 2017; Limame et al., 2012). The biomarkers BGN, AGER and BINP3 have been studied, and their potential in the detection of DKD cannot be overemphasised. However, there are validations, and their limitations may be an obstacle that makes their incorporation into routine testing complicated (Table 1).

**TABLE 1:** Mechanistic roles, diagnostic potential, validation, and limitations of key genes (*BGN*, *AGER*, and *BNIP3*) in renal proximal epithelial tubular injury.

Gene	Role	Biomarker potential	Validation	Limitation
BGN	Extracellular matrix proteoglycan involved in tubulointerstitial fibrosis, inflammation and TGF-β signalling	Increased renal expression in DKD and tubular renal fibrosis	Urinary BGN correlated in a dose-dependent manner with the severity of tubular renal dysfunction (Wyczanska and Lange-Sperandio, 2020)	Conflicting studies and assay variability are affected by age and renal clearance
AGER	Receptor for advanced glycation end products; mediates oxidative stress and inflammation	Extensive mechanistic evidence in diabetes and chronic kidney disease (CKD)	Moderate-strong for soluble RAGE (sRAGE), inconsistent across populations (Steenbeke et al., 2022) (Šebeková et al., 2001)	Variability, affected by age and race
BNIP3	Regulates hypoxia-induced mitophagy, apoptosis and mitochondrial quality control	Strong experimental evidence in renal tubular injury	There are validation studies, however limited, in humans (Hu et al., 2026)	Limited human studies

## Nucleic acid-mediated signalling in tubular epithelial cell injury

Messenger RNA (mRNA) is a single-stranded molecule important in protein synthesis and plays a critical role in RNA metabolism and function (Miyazaki-Anzai et al., 2022). It translates genomic sequences into proteins (Miyazaki-Anzai et al., 2022). mRNA plays a crucial role in the cellular process and phenotype of proximal tubular cells in the kidneys. Single-cell RNA sequencing (RNA-seq) studies have identified specific transcription factors involved in the maturation of tubular epithelial cells.

Furthermore, studies have shown that mRNA levels are altered in various conditions affecting proximal tubular cells. For example, high glucose levels have been found to increase the mRNA expression of BNIP3 in renal tubular epithelial cells (Vaidya et al., 2010). Downregulating BNIP3 can improve cell viability and reduce apoptosis and oxidative stress (Vaidya et al., 2010). Quantitative Reverse transcription polymerase chain reaction (RT-PCR) was used to measure the levels of Sodium-dependent phosphate cotransporter 2A (NPT2A) mRNA, a proximal tubular cell dysfunction marker. Extracellular vesicles (EVs) have also been implicated in regulating mRNA levels in proximal tubular cells (Cao et al., 2025).

Mitochondrial RNA (mtRNA) is RNA transcribed from mitochondrial DNA, which is required for cellular energy and function; cells require their own energy, and thus they possess their own mitochondrial genome in the form of mtRNA. The mtRNA consists of other RNA molecules such as ribosomal RNA, messenger RNA, and transfer RNA.

**FIGURE 3:**
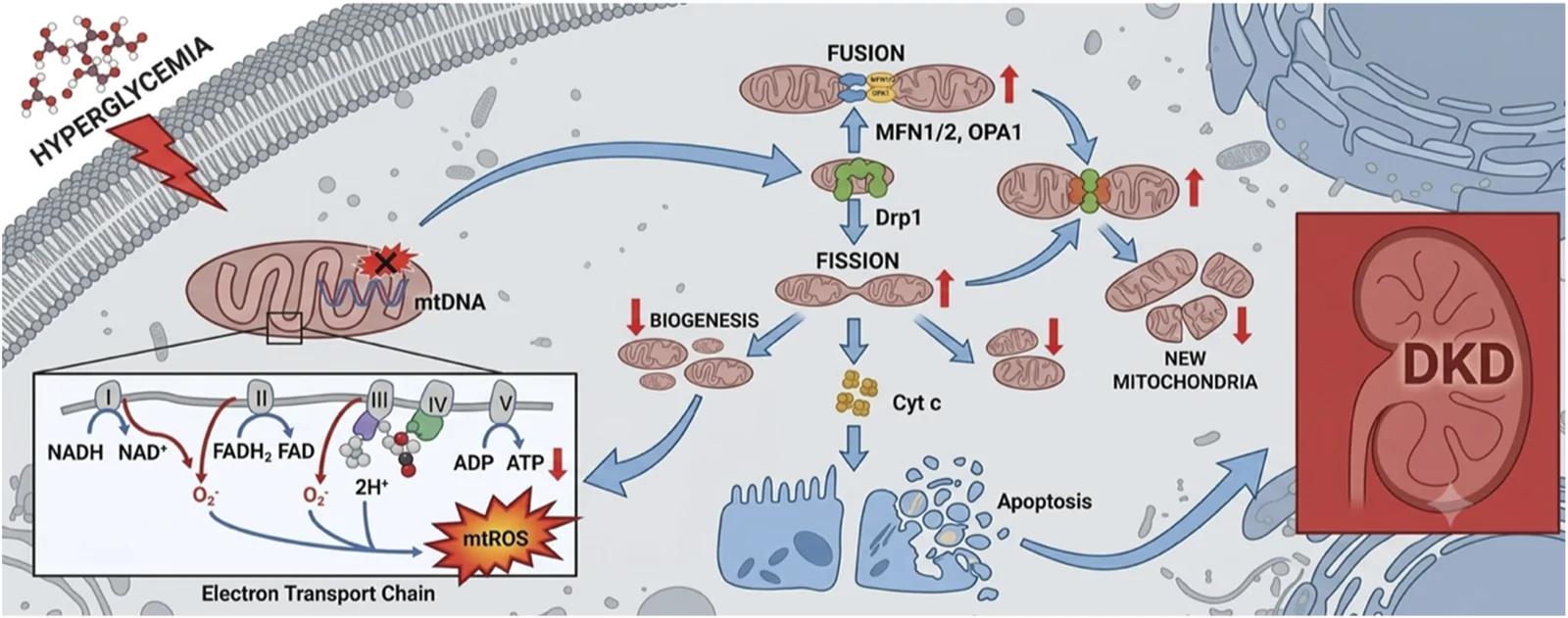
The downstream effect of hyperglycaemia on mtDNA progressing to DKD. Hyperglycaemia damages the mtDNA mtROS through oxidative damage, leading to decreased mitochondrial biogenesis and increased fission, ultimately resulting in renal cell death through apoptosis (ADP: Adenosine diphosphate; ATP: Adenosine triphosphate; FAD/FADH_2_: Flavin adenine dinucleotide (oxidised/reduced forms); NAD/NAD^+^/NADH: Nicotinamide adenine dinucleotide (oxidised/reduced forms).

mtDNA damage is a component in the progression of DKD (Jiang et al., 2019). The irreversible damage to mtDNA by mtROS leads to decreased mitochondrial biogenesis and increased fission, ultimately resulting in renal cell death through apoptosis, contributing to DKD (Jiang et al., 2019). mtDNA is damaged by these reactive processes, contributing to the progression of DKD. Additionally, mtRNA levels may serve as potential biomarkers for functional impairment in DKD (Mauro et al., 2022). The role of hyperglycaemia in diabetes includes the induction of reactive oxygen species (ROS) (Wang et al., 2022), which damage mitochondria and the renal cells that rely on mitochondrial ATP. Additionally, damage to the mitochondrial membrane leads to metabolic dysregulation, characterised by increased ROS and decreased ATP production (Wang et al., 2022). Hyperglycaemia induces damage to renal cells via mitochondrial DNA (mtDNA) damage and the generation of mitochondrial reactive oxygen species (mtROS). This process ultimately leads to diabetic kidney disease (DKD) through the release of cytochrome c (Cyt c), resulting in subsequent apoptosis (Figure 3).

## Diagnostic platforms for characterising transcriptomic and extracellular matrix remodelling in hyperglycaemic PTECs HOLOMONITOR® live cell assay

HoloMonitor® Live Cell Assay (Phase Holographic Imaging AB, Lund, Sweden) is a label-free, quantitative live-cell imaging technique that uses digital holographic microscopy to continuously monitor living cells in culture without fluorescent dyes or stains.

Although the HoloMonitor® technique has been used in various fields such as immunology, oncology, regenerative medicine, stem cells, and *in vitro* drug discovery, its application in kidney cell studies has been limited. However, recent studies have demonstrated the effectiveness of digital holographic cytometry in analysing morphological changes during the epithelial–mesenchymal transition in renal proximal tubule epithelial cells.

Despite the adverse role of hyperglycaemia and diabetes in renal dysfunction, there is still a lack of evidence regarding using the HoloMonitor® imaging technique in monitoring cellular activity, morphology, and wound healing in renal cells. Consequently, incorporating the HoloMonitor® into the diagnosis of hyperglycaemic-induced DKD could yield a better understanding of the effect of hyperglycaemia on the behavioural patterns of renal tubular cells. This platform has been used to address the critical knowledge gap hindered by the limitations of the commonly used transwell migration assay, as it typically functions as an endpoint assay, which limits the ability to capture continuous, comprehensive time-lapse imaging (Justus et al., 2014).

There have been advances in the diagnosis of renal tubular cell dysfunction within a hyperglycaemic microenvironment (Duan et al., 2021). Recent progress has highlighted the potential of novel tubular biomarkers in the screening, diagnosis, and prognosis of DKD, although transitioning these into personalised clinical practice remains a challenge (Duan et al., 2021). This earlier identification can outperform routine renal function tests such as urea, creatinine, and electrolytes (Fiseha and Tamir, 2016), which should not be discarded or ignored, especially in resource-poor regions; however, the combination of molecular techniques and routine tests could be used to achieve a better outcome of identifying early CKD development. These current advances are summarised in Table 2.

**TABLE 2:** Diagnostic platforms for routine tests and characterisation of transcriptomic and extracellular matrix remodelling in hyperglycaemic renal tubular epithelial cells.

Authors	Diagnostic Advance/Feasibility	Year	Diagnostic feature	Sensitivity and specificity	Cost and accessibility
Fiseha and Tamir (2016); Duan et al. (2021)	Urea, creatinine, and electrolytes Standard, routine operational diagnostic workflows	2016–2021	Universally implemented, though they fail to detect early injury compared to molecular techniques	Low sensitivity and specificity; it detects dysfunction when the kidney is 60% damaged	Cheap and economical Widely available; essential baseline for resource-poor regions
Wilson et al. (2019)	Single-cell/single-nucleus RNA sequencing (scRNA-seq/snRNA-seq) applied to diabetic kidney tissue Low for routine care; highly valuable for resolving cell-type-specific mRNA changes and rare cell injury states	2019–2023	Resolves cell-type-specific mRNA changes in proximal tubule and other tubular cells under hyperglycaemia, revealing rare cell states and injury programmes	No validated data on sensitivity and specificity, as this is to support discovery of mechanism	Very expensive and not accessible for routine testing, usually in academic and research laboratories
Polonsky et al. (2024)	Spatial transcriptomics (ST) integrated with scRNA-seq Low maps tissue spatial context to identify localised, tissue-restricted ECM-related gene expression niches	2021–2024	Adds tissue spatial context to transcriptomic changes, identifying localised ECM-related gene expression niches (fibrotic niches, injured PT neighbours)	No validated data on sensitivity and specificity; they are not used in routine clinical pathology laboratories but are used to localise gene expression	Exceptionally high Confined strictly to research setups and advanced kidney disease models
Zhao Y et al. (2020)	Urinary exosomal miRNA profiling High; serves as a noninvasive readout of tubular and epithelial cell mRNA regulation	2020–2023	Noninvasive readout of tubular/epithelial cell mRNA regulation (miRNA cargo mirrors tubular responses)	Sensitivity 75%–95%; specificity 70%–90%. These depend on the miRNA family used (miRNA 21, miRNA 126)	High processing costs Restricted to advanced clinical or research pathology settings
Colhoun, Marcovecchio (2018)	Multiplex urinary biomarker panels (kidney injury molecule 1 (KIM-1), Neutrophil gelatinase-associated lipocalin (NGAL), Liver-type fatty acid-binding protein (L-FABP) High; non-invasive detection of early tubular injury before overt albuminuria develops	2015–2022	Tubular injury markers detectable before overt albuminuria help stratify early tubular involvement in hyperglycaemia	Sensitivity 70%–90%; specificity 65%–88%	Moderate cost Growing utility at the bedside for rapid diabetic patient monitoring
Swaminathan et al. (2022)	High-throughput proteomics/metabolomics (urine and tissue) Excellent for unbiased discovery of altered extracellular matrix (ECM) proteins and secreted marker	2020–2022	Unbiased discovery of ECM proteins and secreted markers altered by hyperglycaemia; candidate panels for early DKD detection	Sensitivity 80%–95%; specificity 75%–90%; it’s an excellent discriminator; however, external validations are limited	High infrastructure costs Minimal access outside of academic research networks
Marshall et al. (2022)	Slide-seqV2/high-resolution spatial methods in kidney disease models Low maps tissue spatial context to identify localised, tissue-restricted ECM-related gene expression niches	2022	Very high-resolution spatial maps of gene expression in kidney; detects spatially restricted tubular injury and ECM gene upregulation	No sensitivity or specificity studies are done, as it's not utilised for clinical diagnostics	Exceptionally high Confined strictly to research setups and advanced kidney disease models
Liu et al. (2023)	Microfluidic/photonic biosensor point-of-care assays for urinary KIM-1/NGAL	2020–2024	Rapid, highly sensitive bedside detection of tubular markers (enables monitoring of tubular damage in diabetic patients)	Sensitivity is high; however, the specificity relies on the biomarker used	Moderate cost Growing utility at the bedside for rapid diabetic patient monitoring
Li Z et al. (2023)	Integrated urine “omics + imaging” diagnostic pipelines (multi-modal diagnostics) Complex pipeline setup requiring full integration of urine panels, exosomal cargo, and tissue mapping	2022–2025	Combining urine biomarker panels, exosomal RNAs, proteomics and spatial/transcriptomic kidney maps to improve sensitivity and mechanistic specificity	Predicted sensitivity: >90% specificity: >85%	Very high cost Currently limited to highly specialised translational medicine consortiums

## Therapeutic targets of hyperglycaemia-induced proximal tubular epithelial cell damage

A hyperglycaemic microenvironment could damage the PTECs via mechanisms such as dedifferentiation, metabolic reprogramming, cell death and senescence and other pathways (ENPP1, HIF stabilisation and epigenetic changes).

The reprogramming and dedifferentiation of PTECs are important in their survival. Surviving PTECs lose their mature markers via dedifferentiation, proliferate and are involved in repair processes through redifferentiation. These processes fail through maladaptive states, which involve fibrosis, inflammation and persistent SOX9+VCAM1+ progenitor cells (Kitai et al., 2025).

Metabolic reprogramming occurs through the mitochondrial oxidative phosphorylation to glycolysis, which are driven by certain factors (GPR87, PFKP, and HK2). Additionally, these lead to the production of histone lactylation, lactate, inflammation and fibrosis. This overall process leads to the exacerbation of injury through mitochondrial dysfunction (Liu and Zhao, 2026).

Regulated cell death through apoptosis, necroptosis and ferroptosis and senescence are contributors to PTEC injury. Additionally, PTEC secrete a senescence-associated secretory phenotype (SASP), an important factor in driving inflammatory processes which eventually leads to fibrosis. Senescent cells are associated with increases in ferroptosis sensitivity (Xu et al., 2025) fuelled by iron accumulation and failed antioxidant defences, eventually leading to cell death.

The upregulation of p21 and p16INK4a is triggered by hyperglycaemia, promoting senescence of tubular epithelial cells, which could promote a pro-inflammatory secretory phenotype (Wang et al., 2023). Additionally, the senescent pathways are therapeutically modulated by targeting kinase-specific pathways, such as the mTOR pathway, which is involved in metabolic reprogramming leading to hypertrophy of the proximal tubular epithelial cells (Mohandes et al., 2023).

There have been known therapeutic targets such as sodium-glucose transporter 2 inhibitors (SGLT2i). SGLT2*i* has a dual function in the modulation and downregulation of the mTORC1 signalling pathway and through the inhibition of renal tubular fibrosis responses mediated by TGF-β (Panchapakesan et al., 2013; Tuttle, 2023). However, beyond the use of SGLT2i, there are NHE3 inhibitors involved in proximal tubular transport and metabolic protection (Wagner, 2024).

The mechanisms above (dedifferentiation metabolic reprogramming, cell death and senescence and other pathways (ENPP1, HIF stabilisation and epigenetic changes)) have led to the novel strategies and therapeutics targets that are part of preclinical studies (2023–2026). These include HIF-prolyl hydroxylase inhibition (e.g., MK-8617), targeting maladaptive Sox9^+^ cells, metabolic targets (glycolysis and mitochondria), senescence and senolytics/senomorphics.

HIF-prolyl hydroxylase inhibition, these are involved in the stabilisation of HIF-1α in PTECs, reprogramming to SOX9^+^ progenitor-like cells and the upregulation of nephrogenesis genes (Li et al., 2025).

The targeting of maladaptive SOX9+ cells improves the health of mitochondrial cells (Dynamin-related protein 1 (Drp1) deletion in SOX9+ cells); this leads to and promotes the reduction of dedifferentiation markers, redifferentiation which stalls the DKD progression. Additionally, the reprogramming of PTECs involves the inhibition of PAX2/8, leading to a less proinflammatory state (Kitai et al., 2025).

Metabolic targets are involved in the inhibition of glycolytic enzymes (PFKFB3, HK2) and MSC-derived exosomes, eventually leading to the inhibition of fibrosis. (Liu and Zhao, 2026). ENPP1 (AD-NP1) reduces scarring and enhances the repair process; this is a promising inhibitor in renal, however, it was originally used in the healing of heart tissue (Iman, 2026).

Senescence and senolytics/senomorphics such as dasatinib + quercetin clear senescent cells and modulate SASP; senescent tubular cells are eliminated by ferroptosis induction (low-dose RSL3) (Liao et al., 2022). Other emerging approaches include reprogramming and regenerative strategies using transcription factor combinations (SIX factors, OCT4) (Omer et al., 2023), and another approach includes the use of nanomedicine and targeted delivery, especially for senescence reprogramming.

Other targets that are noteworthy are the TGF-βR1 inhibitors, IL-11 inhibitors, and cell cycle regulators such as CDK5 and cyclin G1, which are critical in blocking maladaptive repairs without impairing normal repair processes.

## Conclusion

Hyperglycaemia exerts significant adverse effects on renal tubular cells, leading to functional impairments and structural disruptions. Elevated glucose levels enhance the accumulation of reactive oxygen species within tubular epithelial cells and amplify downstream inflammatory injury. Prolonged exposure to a hyperglycaemic microenvironment activates multiple metabolic and signalling pathways, including the polyol and hexosamine pathways, alongside increased formation of advanced glycation end products. These mechanisms trigger a cascade of intracellular events mediated by TGF-β1, NF-κB, and vascular endothelial growth factor, ultimately altering gene expression in proximal tubular cells.

Collectively, these processes illustrate how chronic hyperglycaemia disrupts cellular homeostasis, accelerates fibrotic transformation, and contributes to the progression of diabetic kidney disease. Understanding these mechanisms provides insight into renal tubular dysfunction and highlights potential molecular targets for earlier diagnosis and therapeutic intervention.

Various diagnostic methods are available to screen, diagnose, and monitor renal disease progression. These approaches increasingly integrate advanced imaging with urine biomarker panels, exosomal RNAs, proteomics, and spatial transcriptomic kidney maps to improve sensitivity and mechanistic specificity. Furthermore, evaluating the expression patterns of *BGN* and *AGER* genes may elucidate their involvement in the development and progression of diabetic kidney disease, making them potential candidates for diagnostic panels in the long-term management of diabetic patients.

## Glossary

AGE/AGEs: Advanced glycation end products
AGER: Advanced glycation end products receptor
α-SMA: α-smooth muscle actin
ADP: Adenosine diphosphate
ATP: Adenosine triphosphate
BGN: Biglycan
BNIP3/BINP3: BCL2 interacting protein 3
CKD: Chronic kidney disease
Cyt c: Cytochrome c
DKD: Diabetic kidney disease
Drp1: Dynamin-related protein 1.
ECM: Extracellular matrix
EMT: Epithelial–mesenchymal transition
EV/EVs: Extracellular vesicle/Extracellular vesicles
**FAD/FADH**_**2**_: Flavin adenine dinucleotide (oxidised/reduced forms)
HMGB1: High-mobility group box 1
hnRNA: Heterogeneous nuclear ribonucleic acid
IL-1α: Interleukin-1 alpha
KIM-1: Kidney injury molecule 1
L-FABP: Liver-type fatty acid-binding protein
MAP/MAP kinase/MAPKKK: Mitogen-activated protein/Mitogen-activated protein kinase/Mitogen-activated protein kinase kinase kinase
MKK6: Mitogen-activated protein kinase kinase 6
mRNA: Messenger RNA
mtDNA: Mitochondrial DNA
mTOR/mTORC1: Mammalian target of rapamycin/Mammalian target of rapamycin complex 1
mtRNA: Mitochondrial RNA
mtROS: Mitochondrial reactive oxygen species
**NAD/NAD** ^**+**^ **/NADH**: Nicotinamide adenine dinucleotide (oxidised/reduced forms)
NF-κB: Nuclear factor-κB
NGAL: Neutrophil gelatinase-associated lipocalin
NPT2A: Sodium-dependent phosphate cotransporter 2A
pEMT: Partial epithelial-mesenchymal transition
PI3K/PI3K/Akt: Phosphoinositide 3-kinase/Phosphoinositide 3-kinase and protein kinase B signalling pathway
PKC: Protein kinase C
PTEC/PTECs: Proximal tubular epithelial cell/cells
PT: Proximal tubule
RAGE/sRAGE: Receptor for advanced glycation end products/Soluble receptor for advanced glycation end products
RNA-seq/scRNA-seq/snRNA-seq: RNA sequencing/single-cell RNA sequencing/single-nucleus RNA sequencing
ROS: Reactive oxygen species
RT-PCR: Reverse transcription polymerase chain reaction
RUNX1: Runt-related transcription factor 1
SGLT2/SGLT2i: Sodium-glucose transporter 2/Sodium-glucose transporter 2 inhibitors
SLRP: Small leucine-rich repeat proteoglycan
ST: Spatial transcriptomics
TGF/TGF-β/TGF-β1: Transforming growth factor/Transforming growth factor-β/Transforming growth factor-beta 1
TLRs: Toll-like receptors
VEGF: Vascular endothelial growth factor
Wnt/β-catenin: Wingless and Int-1/beta-catenin signalling pathway
